# A Study on the Cement Gel Formation Process during the Creation of Nanomodified High-Performance Concrete Based on Nanosilica

**DOI:** 10.3390/gels8060346

**Published:** 2022-06-02

**Authors:** Alexey N. Beskopylny, Sergey A. Stel’makh, Evgenii M. Shcherban’, Levon R. Mailyan, Besarion Meskhi, Valery Varavka, Nikita Beskopylny, Diana El’shaeva

**Affiliations:** 1 Department of Transport Systems, Faculty of Roads and Transport Systems, Don State Technical University, Gagarin, 1, 344003 Rostov-on-Don, Russia; 2 Department of Engineering Geology, Bases, and Foundations, Don State Technical University, 344003 Rostov-on-Don, Russia; sergej.stelmax@mail.ru (S.A.S.); au-geen@mail.ru (E.M.S.); 3 Department of Roads, Don State Technical University, 344003 Rostov-on-Don, Russia; lrm@aaanet.ru; 4 Department of Life Safety and Environmental Protection, Faculty of Life Safety and Environmental Engineering, Don State Technical University, Gagarin, 1, 344003 Rostov-on-Don, Russia; spu-02@donstu.ru; 5 Research and Education Center “Materials”, Don State Technical University, Gagarin sq., 1, 344003 Rostov-on-Don, Russia; varavkavn@gmail.com; 6 Department Hardware and Software Engineering, Don State Technical University, 344003 Rostov-on-Don, Russia; beskna@yandex.ru; 7 Department of Technological Engineering and Expertise in the Construction Industry, Don State Technical University, 344003 Rostov-on-Don, Russia; diana.elshaeva@yandex.ru

**Keywords:** cement gel, nanomodified high-performance concrete, nanosilica, concrete composition

## Abstract

One of the most science-intensive and developing areas is nano-modified concrete. Its characteristics of high-strength, high density, and improved structure, which is not only important at the stage of monitoring their performance, but also at the manufacturing stage, characterize high-performance concrete. The aim of this study is to obtain new theoretical knowledge and experimental-applied dependencies arising from the “composition–microstructure–properties” ratio of high-strength concretes with a nano-modifying additive of the most effective type. The methods of laser granulometry and electron microscopy are applied. The existing concepts from the point of view of theory and practice about the processes of cement gel formation during the creation of nano-modified high-strength concretes with nano-modifying additives are developed. The most rational mode of the nano-modification of high-strength concretes is substantiated as follows: microsilica ground to nanosilica within 12 h. A complex nano-modifier containing nanosilica, superplasticizer, hyperplasticizer, and sodium sulfate was developed. The most effective combination of the four considered factors are: the content of nanosilica is 4% by weight of cement; the content of the superplasticizer additive is 1.4% by weight of cement; the content of the hyperplasticizer additive is 3% by weight of cement; and the water–cement ratio—0.33. The maximum difference of the strength characteristics in comparison with other combinations ranged from 45% to 57%.

## 1. Introduction

Currently, in modern construction, there is a problem associated with the mismatch of existing traditional building materials with the ever-growing high rate of requirements for buildings and structures. There are problems that relate to territory preparation, problems of complex engineering and geological conditions, differences in the density of urban development, and many other complicating factors that ultimately lead to a large overspending of resources, materials, energy, labor, and time during the construction of buildings and structures.

One of the most popular materials used in modern construction is concrete. It is used both for traditional reinforced concrete products and structures and various types of monolithic concreting. At the same time, concrete, remaining one of the most popular applied materials, is also the subject of study and research by many scientists involved in developments in the field of construction.

Therefore, one of the most promising concrete types is high-strength concretes. These concretes, class B60 and higher in strength, can reach even more significant values when composed correctly and when the correct technological factors are applied in their manufacture. The class of concrete is the value of the cubic compressive strength of concrete in MPa, which is provided with a confidence probability of 0.95, considering the statistical variability of concrete strength. One of the most science-intensive and developing areas is nanomodified concrete. High-strength concretes are characterized not only by high-strength characteristics, but also by high density and improved structure, which is important to ensure not only at the stage of monitoring their performance, but most importantly, at the stage of their manufacture.

### 1.1. Literature Review

The prior knowledge required to study building materials, their properties, characterization methods, and standard laboratory test methods is provided in [1]. The use of innovative, new, and recycled materials in civil engineering is of great importance to produce high-quality, improved cementitious composites. A knowledge of these materials, including their origin, formation, physical and mechanical properties, and decay behavior, plays a major role in the selection, handling, use, maintenance, and recycling of these materials [1]. Currently, the design of pavements by a mechanistic method is a key area of research [2,3,4,5].

Slag nanomodification of cement composites has already established itself as a promising and effective solution for improving the micro- and macrostructure of various characteristics of concretes, mortars, products, and structures composed of them [6,7,8,9,10,11]. At the same time, much attention is being paid to the parameters of slag grinding; its activation, including alkaline; its use in combination with other finely dispersed additives, for example, fly ash and nanosilica; as well as the processes of hydration and structure formation of Portland slag cement [9].

Ash, similar to slag, is also an effective additive in concrete, used as a replacement for a part of cement, or in the form of a nanomodifying additive, improving the compressive and flexural strength, plasticity, and deformation characteristics [12,13,14,15,16,17,18,19,20,21] of cement compositions, which, in microstructural studies, present a denser gel matrix and a stronger bond in the interfacial transition zone [13]. It should be noted that the maximum effects from the use of fly ash in various types of concrete are possible, including its use together with other additives, such as microsilica [15,19], nanosilica [12,13,18,19], calcium nano-carbonate [12,14,20,21], crumb rubber [15,18], and C-S-H nanoparticles [17].

A common method for obtaining high-strength concretes is the addition of carbon nanotubes (CNTs) or multilevel carbon nanotubes (MWCNTs) [22,23,24,25,26]. CNTs modify the failure process in the cement matrix where microcracks are initiated with “significantly improved fracture energy resulting in improved global mechanical properties and a nanosized interfacial transition zone is found which determines the failure of the nanocomposite system” [22]. In composites with CNTs, fluctuations in the degree of polymerization occur depending on the particle size, crystalline phases, and surface modification of the nanofillers [24]. The addition of a small amount of carbon nanotubes with nanosilica, nano-alumina, and nanoclay results in the maximum strength development of geopolymer concrete. The inclusion of these nanomaterials in the geopolymer significantly improves the structural stability, increasing its durability [25]. Modification of ordinary Portland cement with a suspension of multi-walled carbon nanotubes (MWCNT) with microsilica provided “strong adhesion of additives and cement hydration products” [26].

The modification of the following types of additives proved to be effective in improving the characteristics of cement composites: flask, diatomite [27], ultrafine quartz [28], nanomethakaolin [29], nanotitania [24,27,30], recycled aggregate [23], slaked lime [13], rice husk ash [31], nanosized cellulose filaments [32], nanofluid complex additive (graphene/PVA) [33], boron nanonitride, multilayer graphenes, zirconium oxide [24], nano-aluminum oxide [25,34], nanoclay [25], and iron and lead nano-oxides [35].

The investigation of calcium silicate hydrate gel, which is the main binder phase in Portland cement, at the micro- and nanoscales allows us to identify and quantify the strength scaling “mechanisms arising from various types of defects, and critically assesses the susceptibility of CSH gel to physical and/or chemical wear, quantifies determining their influence on the structural integrity of the gel, which is subsequently projected as a decrease in the bearing capacity of the material” [36]. The mechanisms of “the effect of nanofillers on the C-S-H gel are explained by the nucleation effect and the pozzolanic effect (for nano silica) of nanofillers that promote cement hydration; high water absorption capacity of nanofillers, which reduces the proton water inside the C-S-H gel and reduces the distance between the structural groups of Ca, O, and Si atoms” [24]. The addition of CSH nanoparticles as nuclei has been successfully used to accelerate the process of the hydration and settling of binder phases in both conventional Portland cement and alternative binders [17,37]. In addition, CSH gels are known as the most widely used synthetic materials [38].

Modification with microsilica is currently very popular due to its effectiveness in improving the properties of concrete both separately and in combination with other additives [15,19,26,27,28,39,40,41,42,43]. The most effective dosages of microsilica vary from 5 to 20%, depending on the raw materials used and the types of concrete. At the same time, the increase in strength characteristics varies from insignificant, when only microsilica is added, to quite impressive (30–80%), when other additives are used along with microsilica, in particular, flask and diatomite [27], fly ash [19], carbon nanotubes [26], nanosilica, and nanosilica sol [19,41,43,44]. Microsilica densifies and strengthens the structure, increasing its uniformity and stabilizing the cement hydration process [19,27,40,41,42,43,44].

The nano-modification of concrete with nanosilica [12,13,18,19,23,24,25,35,41,43,44,45,46,47,48,49,50,51] has already shown efficiency in improving the properties of concrete, but still requires a solution for many issues. The main problem associated with the introduction of nanoparticles into cement-based materials is how to evenly distribute them in the matrix [23]. The interaction of nanosilica with other additives [12,13,18,19,23,24,25,34,35], as well as the features and mechanisms of its effect on the C-S-H gel [24], also require an even more complete study. The use of microsilica with fly ash shows a denser gel matrix and a stronger bond in the interfacial transition zone in all age groups [13]. The addition of nanosilica to other nano-additives significantly increases the strength of geopolymer concrete, improves structural stability, and increases its durability [25]. An important problem of the nanomodification of cement composites is the efficiency of dispersion of nanomaterials inside the cement matrix. In [35], the influence of the type and size of nanoparticles on their tendency to agglomerate in the cement matrix is considered. Dispersion methods included the use of high-speed mechanical mixing and ultrasonic treatment [35]. Compared to unmodified nanosilica, silane binder-modified nanosilica promotes and accelerates the cement hydration process. The “pozzolanic effect, filling effect, and nucleation effect of the modified nano silica made the microstructure of the composite more compact and thus improved the static mechanical properties of cement-based composites” [49].

Article [31] describes the conditional ways of establishing the relationship between the degree of hydration (non-evaporating water) and porosity between the gel/space ratio and the compressive strength of a solution from a high-strength mixture at an early age. Using the non-evaporating water method, the gel/space ratio of solidified HPC solutions was predicted. The results of this study show that the addition of gel-like calcined clay and rice husk ash (5–30% by weight of Portland cement) to the composite matrix leads to a moderate decrease in the compressive strength of a HPC solution with a low water–binder ratio (W/B) [31].

### 1.2. Theoretical Prerequisites for Obtaining High-Strength Nano-Modified Concretes

**Figure 1:**
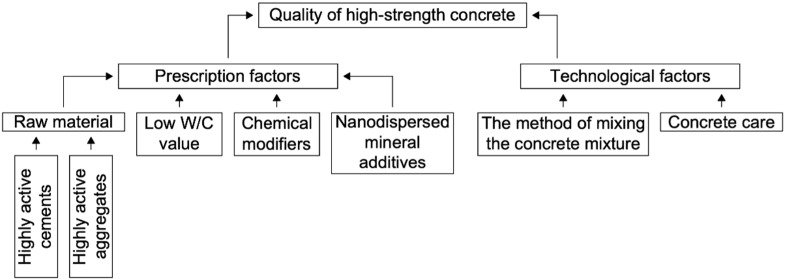
The main factors affecting the final value of the physical and mechanical characteristics of high-strength concrete.

In general, in the technology of high-strength concrete, it is important to take into account a number of factors that guarantee the creation of a strong and dense concrete structure with a correspondingly high final strength (see Figure 1).

It has been established that the physical and mechanical properties of cement stone and concrete as a whole are affected by the ratio between the crystalline and gel-like phases, as well as the presence of ultrafine filler, including nanoparticles. With the optimal ratio between the gel-like and crystalline components of the composite mass, a denser packing of filler grains is provided [52].

The most promising direction in the creation of nano-modifying additives is the development of complex nano-modifiers, for which new-generation superplasticizers are used in combination with various nanosized active and low-active mineral additives. To control the processes of structure formation of cement systems, it is necessary to synthesize such organomineral substances, which, depending on the structure of the inorganic carrier and the organic component, the degree of modification of the sorbent and the strength of the fixation of organic substances on the surface of the solid phase will be able to split off the organic substance in the required technological period and thereby provide a differential impact on the rheological properties, rate of hydration, and localization of neoplasms [53].

The achievement of the maximum characteristics of high-strength concretes is due to the effectiveness of the superplasticizers used, which ensure the production of workable mixtures at the lowest possible values of the water–cement ratio. In this study, the optimization of the compositions of nano-modifiers was conducted. The “influence of sulfate-containing additives such as sodium sulfate is reduced to accelerating the hydration of the silicate phases of cement by increasing the ionic strength of the solution. In addition, the exchange reactions of the additive anion with the portlandite phase (Ca(OH)_2_) and aluminate phases of the hardening cement have a significant effect on hardening, which leads to the formation of easily soluble hydroxides and sparingly soluble calcium salts” [53].

### 1.3. The Mechanism of Influence of Nano-Modifiers on the System of Hardening High-Strength Fine-Grained Concretes

The influence of nanoscale modifiers on the hardening system of high-strength fine-grained concretes can be explained as a result of the interaction of several mechanisms:

- Nanosized particles provide an increase in the volume of chemisorption-bound water and a decrease in capillary-bound water; all this together leads to an increase in the packing density of dispersed particles and a general decrease in the porosity of the cement matrix;
- The catalytic role of nanosized particles as crystallization centers with the corresponding effect of lowering the energy threshold of this process and accelerating it;
- Nanosized particles participate in the chemical processes of the phase formation of hydrated compounds.

Therefore, for example, the implementation of the first mechanism depends directly on the physical characteristics of nano-additives (granulometric composition and specific surface area) and their dosage.

The catalytic mechanism is realized at the stages of colloidation, nucleation, and phase formation, when nanosized particles act as crystalline seeds and crystallization centers.

A chemical interaction can be performed only under the condition that the composition of the particles substantially corresponds to the products of hydration of cement minerals [54].

### 1.4. Novelty, Purpose, and Objectives of the Study

Thus, there is a scientific deficit, to which the research will be directed, as well as to the solution of applied problems of modern construction, described above.

Due to the fact that the subject of the study is not only the characteristics of the finished concrete, but also the process of its structure formation, we set the following research objectives:

- Firstly, the study of the process of cement gel formation during the creation of nano-modified high-strength concretes with nano-modifying additives;
- Secondly, we choose the most rational way of performing the nano-modification of such concretes through additional research in terms of optimization;
- Thirdly, and finally, by linking the well-known combination of “composition–structure–properties” in relation to nano-modified concrete, we show the applied effectiveness of the theoretical knowledge obtained for the structure formation and properties of high-strength concrete using nano-modifiers.

The aim of the study is to obtain new theoretical knowledge and experimental-applied dependencies arising from the “composition–microstructure–properties” ratio of high-strength concretes with a nano-modifying additive of the most effective type.

## 2. Materials and Methods

### 2.1. Materials

Portland cement grade PC 500 D0 (CEM I 42.5 N) produced by OAO Novoroscement was used as a binder in the experimental studies. The main characteristics and chemical and mineralogical compositions of the cement are presented in Table 1 and Table 2. Data for the chemical and mineralogical compositions were obtained from the manufacturer from the cement-quality certificate.

Quartz sand produced by OAO Arkhipovsky Quarry (Arkhipovskoe village, Russia) was used as a fine aggregate. Fine aggregate characteristics, according to GOST 8736–2014 “Sand for construction works. Specifications”, are presented in Table 3. Each sand test was performed in accordance with GOST 8735-88 “Sand for construction work. Testing methods”.

Microsilica grade MK-85 was used as a nano-modifying additive. The chemical composition, granulometric characteristics, and X-ray diffraction analysis of microsilica are presented in [55,56,57].

To regulate the processes of structure formation of concrete mixtures and the physical and mechanical properties of concrete, various chemical additives were introduced into the composition of the molding mixtures: superplasticizer MELFLUX 1641 F BASF Construction Additives (Krasnodar, Russia); hyperplasticizer “MC-PowerFlow” (“MC-Bauchemie”, St. Petersburg, Russia) based on the latest technology of MS polycarboxylate esters; and sodium sulfate MetallEnergoHolding group of companies (Irkutsk, Russia). The technical characteristics of the additives are presented in Table 4, Table 5 and Table 6. Test methods for sodium sulfate comply with GOST 21458-75 “Crystallization sodium sulfate”.

### 2.2. Methods

The grinding of microsilica MK-85 was performed in a planetary ball mill “Activator-4M” (Novosibirsk, Russia) for 2, 4, 6, 8, 10, 12, and 14 h at a speed of 800 rpm.

For the manufacturing and further testing of samples in this study, the following equipment was used:

- Technological equipment—laboratory concrete mixer BL-10 (ZZBO LLC, Zlatoust, Russia);
- Testing equipment—hydraulic press IP-1000 (NPK TEHMASH LLC, Neftekamsk, Russia), R-50 tensile testing machine (IMash LLC, Armavir, Russia);
- Measuring instruments [58,59,60,61,62,63,64].

The structure of the modified cement stone was studied using a ZEISS CrossBeam 340 double-beam scanning electron/ion microscope equipped with an Oxford Instruments X-Max 80 X-ray microanalyzer (Carl Zeiss Microscopy GmbH (Factory), Jena, Germany)” [58].

The study of the granulometric characteristics was conducted using a CPS Disk Centrifuge DC24000 centrifuge (CPS Instruments Europe, Oosterhout, The Netherlands). The CPS centrifuge is a fast and sensitive particle-size analyzer for colloidal solutions. This instrument works with particles ranging in size from 10 nm to 40 µm.

The amount of binder and sand for the preparation of the experimental compositions of high-strength fine-grained concrete remained unchanged. The consumption of cement was always 760 kg per 1 m^3^ of concrete mix, and the consumption of fine aggregate was 1175 kg per 1 m^3^ of concrete mix. The content of the sodium sulfate additive also remained unchanged and amounted to 1.5% by weight of the binder.

To achieve the maximum effect of modification with a complex additive, the following procedure for preparing a fine-grained concrete mixture was adopted. To a thoroughly mixed mixture of aggregate, Portland cement and sodium sulfate with the addition of the superplasticizer MELFLUX 1641 F, a part of the mixing water (85% of the total consumption) was added. Separately, in the Activator-4M planetary ball mill, a complex nano-modifier was prepared in the form of a suspension, consisting of a mineral additive (nanosilica), the MC-PowerFlow hyperplasticizer, and the rest of the mixing water. Subsequently, the finished nano-modifier in the form of a suspension was added to the concrete mixture and mixed for 5 min. With such a scheme for preparing a concrete mixture, competitive adsorption of two superplasticizers for positively charged active functional groups on the surface of the solid phase was excluded. In this case, the superplasticizer introduced into the mixture of aggregate with Portland cement paste was adsorbed on particles of clinker minerals and hydrate neoformations, dispersing the formed floccules [53].

Ready-mixed concrete was poured into metal molds (Smolenskoe SKTB SPU JSC, Smolensk, Russia) and vibrated on a laboratory vibration platform SMZh-539-220A (IMASH LLC, Armavir, Russia). Then, the molds with the mixture were placed in a normal hardening chamber (RNPO RusPribor LLC, St. Petersburg, Russia) and kept there for a day. Subsequently, the samples were removed from the molds and again placed in the hardening chamber, where they were kept for another 27 days. Following 28 days, the samples were subjected to strength tests according to GOST 10180 “Concretes. Methods for strength determination using reference specimens”.

**Figure 2:**
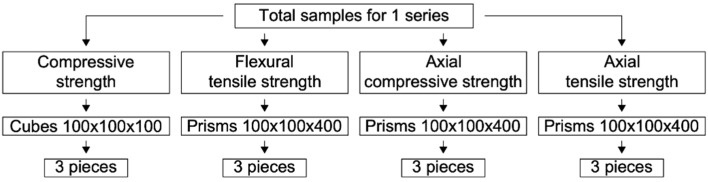
Experimental research program.

The experimental research program is shown in Figure 2.

## 3. Results

### 3.1. Development of a Method for Obtaining Nanosilica by Mechanical Dispersion in a Planetary Ball Mill “Activator-4M”

To determine the optimal conditions for the mechanical dispersion of microsilica to a nanoscale state, several experiments were conducted. The grinding of microsilica was performed at a speed of 800 rpm.

**Figure 3:**
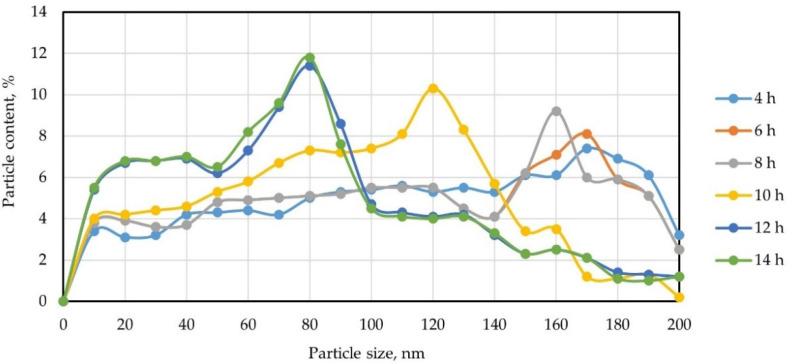
Size distribution of nanosilica particles at different grinding times.

The size distribution of microsilica particles following grinding is shown in Figure 3. The dispersion of microsilica particles for 2 h turned out to be ineffective: the proportion of nanosized particles (<100 nm) was 12%.

Note that, with an increase in the grinding time, significant changes in the particle-size distribution can be observed. Thus, for silica fume subjected to mechanical activation for 4 h, the maximum peak falls on particles 170 nm in size and amounts to 7.4%; with the increase in the dispersion time reaching 8 h, the maximum peak of the distribution of the particles shifts to the left, which corresponds to particles with a size of 160 nm and is 9.2%. The same is true for particles ground for 10 h; however, here, a sharper jump is observed towards a decrease in particle dispersion. Here, the peak falls on particles 120 nm in size and amounts to 10.3%. When microsilica is activated for 12 h, the peak falls on particles of 80 nm (11.4%); that is, the jump in the decrease in dispersion is comparable to a grinding time of 10 h. However, with an increase in the grinding time to 14 h, the peak, as well as at 12 h, falls on particles with a size of 80 nm and amounts to 11.8%. From all this, we can conclude that the most effective mode of mechanical dispersion of microsilica is its grinding for 12 h at a speed of 800 rpm.

### 3.2. Optimization of the Composition of the Nano-Modifier According to the Criteria of Strength Characteristics

The optimization of the composition of the nano-modifier was performed using the Box–Wilson second-order orthogonal compositional planning method.

The optimization parameters and values of the variation factors are shown in Table 7 and Table 8, respectively.

The results for determining the strength characteristics of fine-grained high-strength concretes modified with a nano-additive are presented in Table 9.

The significance of the coefficients of the regression equation was checked using the Student’s *t*-test, and the adequacy of the regression equations for the experimental data was checked using the Fisher criterion. Thus, according to the results of the calculations, the following regression Equations (1)–(4) were obtained:

(1) $$R_{b.cub}=96.866-6.284\cdot X_{1}^{2}-6.745\cdot X_{2}^{2}-6.448\cdot X_{4}^{2}\;(R^{2}=0.841)$$

(2) $$R_{b}=72.657-4.566\cdot X_{1}^{2}-5.301\cdot X_{2}^{2}-4.942\cdot X_{4}^{2}\;(R^{2}=0.884)$$

(3) $$R_{btb}=11.684-6.284\cdot X_{1}^{2}-6.745\cdot X_{2}^{2}-6.448\cdot X_{4}^{2}\;(R^{2}=0.911)$$

(4) $$R_{bt}=7.266-0.534\cdot X_{1}^{2}-0.566\cdot X_{2}^{2}-0.541\cdot X_{4}^{2}\;(R^{2}=0.928)$$

It follows from the data in Table 9 that the most significant influence on the strength characteristics of high-strength fine-grained concretes is exerted by the following factors: nanosilica dosage; dosage of superplasticizer MELFLUX 1641 F; and water–cement relationship.

Additionally, the most effective combination of all four studied factors is:

- The content of nanosilica—4% by weight of cement;
- Content of superplasticizer additive MELFLUX 1641 F—1.4% by weight of cement;
- The content of the hyperplasticizer additive “MC-PowerFlow”—3% by weight of cement;
- Water–cement ratio—0.33.

**Figure 4:**
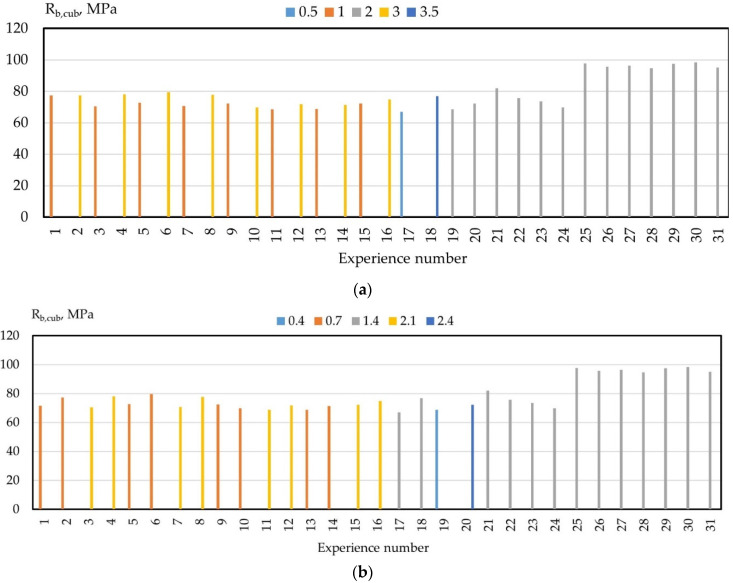

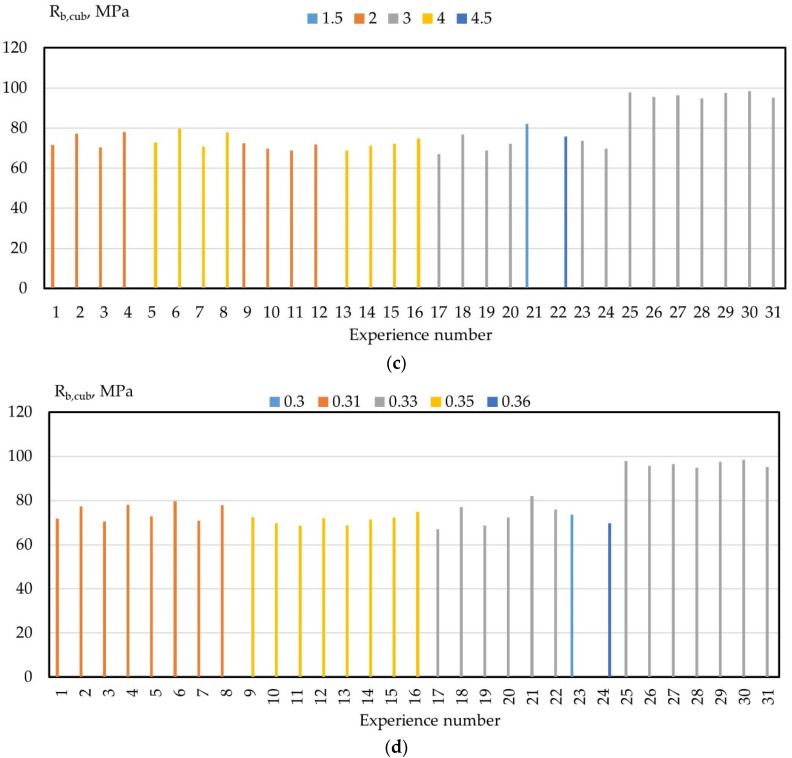
Dependence of the compressive strength of high-strength concretes on factors: (**a**) X_1_; (**b**) X_2_; (**c**) X_3_; and (**d**) X_4_.

To illustrate this statement, let us present the influence of each of the factors on the change in the compressive strength of nano-modified high-strength fine-grained concrete (Figure 4).

A similar pattern of change is also observed for the axial compressive strength, tensile strength in bending, and axial tensile strength.

The introduction of a nano-modifying additive based on silica makes it possible to eliminate the defectiveness of the concrete structure by filling the microcracks and micropores of fine-grained concrete with nanosilica and products of its interaction with cement clinker minerals [54].

**Figure 5:**
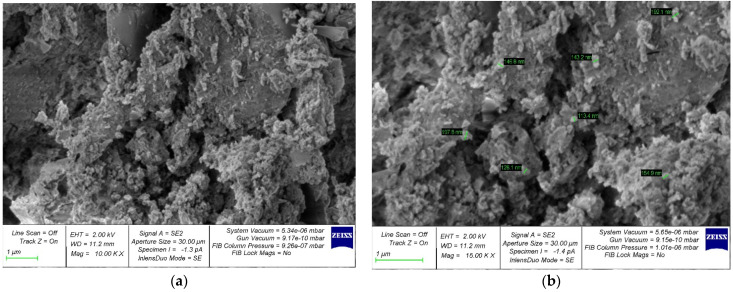
Samples with nanosilica content of 2.5% by weight of cement at magnifications of (**a**) 10,000× and (**b**) 15,000×.

**Figure 6:**
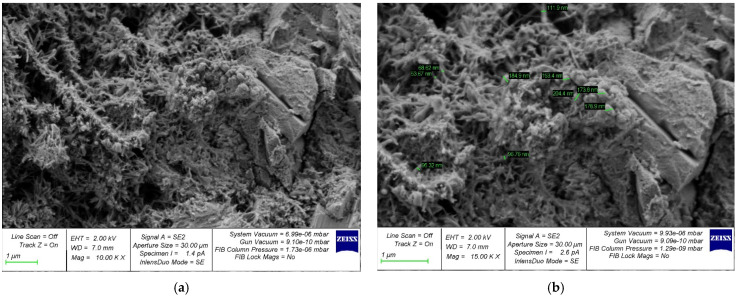
Samples with a nanosilica content of 3% by weight of cement with an increase: (**a**) 10,000× and (**b**) 15,000×.

**Figure 7:**
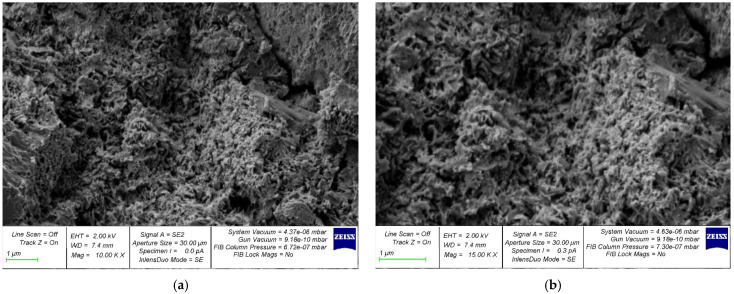
Samples with a nanosilica content of 4% by weight of cement with an increase: (**a**) 10,000× and (**b**) 15,000×.

**Figure 8:**
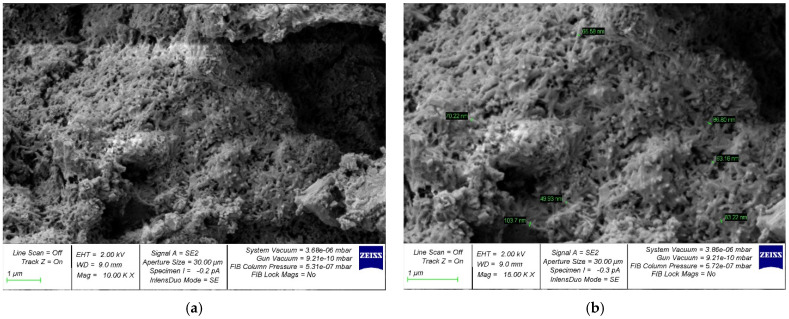
Samples with a nanosilica content of 5% by weight of cement with an increase: (**a**) 10,000× and (**b**) 15,000×.

**Figure 9:**
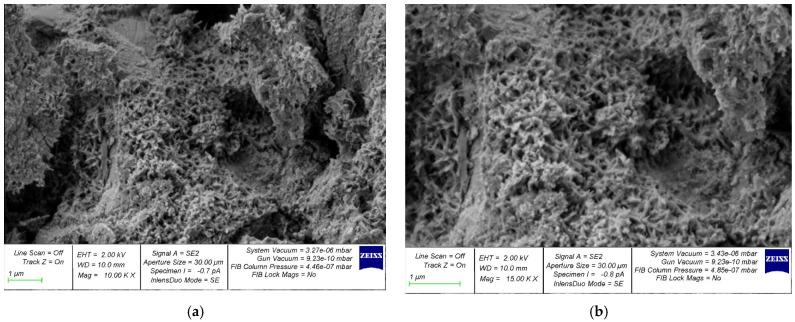
Samples with a nanosilica content of 5.5% by weight of cement with an increase: (**a**) 10,000× and (**b**) 15,000×.

The structure formation of a cement composite nano-modified by the developed complex nano-modifier based on nanosilica was studied using photographs of the microstructure, which are shown in Figure 5, Figure 6, Figure 7, Figure 8 and Figure 9.

In Figure 5a, Figure 6a, Figure 7a, Figure 8a and Figure 9a, at a 10,000× magnification, microdefects can be observed in the areas where microcracks begin to form in the structure of the cement composite. In Figure 5b, Figure 6b, Figure 7b, Figure 8b and Figure 9b, at a magnification of 15,000×, it is already possible to consider nanomodifying additives, places in which the structure is compacted as a result of them, and the appearance of new nanomodified crystallization centers. This scale of photographs of the microstructure allows us to more fully analyze the picture of the nanomodification of the cement composite with nanosilica particles.

It is clear, according to the test data for a physical experiment, the optimal dosage of nanosilica in high-strength concrete has a parabolic dependence and there are cases of both the undersaturation of concrete with a nano-modifier, which leads to a deterioration in its structure and properties, and possible cases of the oversaturation of concrete with nanosilica, which also reduces its characteristics. and degrades its structure. This can be clearly observed on the microphotographs, in which, at the optimal dosage of nanosilica, the densest structure is observed with the densest packing of particles, the smallest number of microstructural defects, and, in general, the relationship of “the most perfect structure—the best properties” is also clearly observed (Figure 7). In the case of undersaturation at dosages of 2.5% (Figure 5) and 3% (Figure 6), we can observe an understaffed structure due to insufficiently dense packing with many vacuum pours, which, in turn, leads to the ratio of “insufficiently perfect microstructure–insufficiently improved properties”. As for the case of the supersaturation of high-strength concrete with a nano-modifier, its structure acquires an imperfect form (Figure 8 and Figure 9). The lack of a sufficient number of crystallization centers is due to an increase that exceeds the optimal content of the nano-modifier, which does not fully reveal the mechanism for improving the structure and properties due to a rationally selected modification technology. Thus, it is important to observe the optimal dosage of the nano-modifier. It is in this case that the “microstructure–properties” ratio is the most harmonious.

Having studied the photo of the microstructure of the resulting composite, based on which the nano-modified high-strength concrete on nanosilica is created, we presented a theoretical interpretation and textual description of the obtained photographs of the microstructure. As is known, in traditional concrete without the presence of nano-modifiers, the process of cement hydration is important, and crystallization during concrete-structure formation occurs according to a typical standard scenario with the gradual disappearance of physically bound water from concrete, which accompanies cement hydration and the curing of the concrete during the process. The nano-modifier used by us allowed us to intervene and control the process of the formation of new crystallization centers due to the active interaction between nanosilica particles, provided that these particles were rationally dosed, which allowed new crystallization centers to form in the concrete body and contribute to the formation of a denser packing of particles of an improved structure and, ultimately, greater concrete strength. In this case, we acted not only at the level of physically bound water, but also influenced the process of cement gel formation due to the appearance of new nano-modified crystallization centers.

## 4. Discussion

Earlier in the studies [50], we obtained data for improving the structure formation and properties of new concrete. In the previous study, we considered microsilica as a nano-modifier, which already at its level produced a high performance and led to an increase in strength and other physical and mechanical characteristics of traditional concrete based on Portland cement. In the current study, unlike the previous one, we studied the effect of an even more effective addition of a nano-modifier based on nanosilica on the formation of structure-formation processes and properties, considering the very mechanism and physical essence of cement gel formation at the microscopic level. The ability to intervene and control the formation of cement gel and chemically bound water was established.

In this regard, our current research has led to even better results and made it possible to control the structure and properties of already high-strength concretes, the characteristics of which increase depending on the improvement of the structure due to the method of the nano-modification of high-strength concretes with the addition of nanosilica that we used.

**Figure 10:**
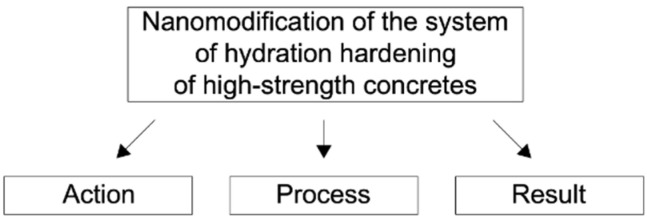
Aspects of nano-modification of hydration-hardening systems for high-strength concretes.

The process of the nano-modification of high-strength concretes can be considered in three aspects (Figure 10).

The “Action” aspect can be considered in an effort to perform the “Process” of cement hydration and cement-stone-structure formation to obtain the desired result. Therefore, for example, the effectiveness of the use of complex modifying additives can be assessed according to three criteria: *E* is the energy efficiency of the process of composite-structure formation; *τ* is the duration of the process of structure formation of the composite; and *R* is the quality of the resulting composite, namely, its physical and mechanical characteristics.

Quantitative and qualitative kinetic changes in the characteristics of the solid–liquid phases and the pore space of hardening cement are the result of the sequential and parallel-sequential flow of the following processes in time: wetting, adsorption, and chemisorption; peptization of cement particles; dissociation of cement clinker minerals and formation of corresponding cations and anions; diffusion of dissociation products from the “surface” to the “intergranular” hydration volume; formation of and changes in the course of diffusion of the concentration gradient of these products in the “surface” and “intergranular” volumes; a gradual increase in the concentration of anions and cations; and the achievement of the state of their saturation and supersaturation in the liquid phase of the “intergranular” volume [54,65].

Thus, the assessment of the effectiveness of nano-modification according to criteria E and *τ* can be conducted by applying the function of the degree of cement hydration in time *V_h_ = f*(*τ*), the rate of hydration *dV_h_/dτ*, the activation energy of process *E*_a_ in a causal correlation of these indicators with the type of nano-modifier additives and their dosage.

The means of controlling the process of cement hydration should first be considered, such as the change in the conditions of saturation and supersaturation of the “water + cement” system with anions and cations. Changing the conditions of saturation and supersaturation usually depends on the rate of hydration and the rate of the diffusion of cations and anions in relation to the “intergranular” volume.

The greater the supersaturation created in the system, the lower the energy threshold for the formation of particles of a new phase. At the same time, the higher the saturation, the smaller the critical size of nascent particles [54,66].

The effectiveness of introducing additives of nano-modifiers can, based on the foregoing, be characterized by a measure of lowering the energy threshold for the formation of particles of a new phase and, accordingly, decreasing the activation energy *E*_a_ of the hydration process. Its velocity constant *K* increases exponentially (according to Arrhenius), according to

(5) $$K=A\cdot \exp\left(-\frac{E_{a}}{RT}\right)$$

where *A* is the pre-exponential factor (frequency factor) characterizing the frequency of collisions of reacting molecules; *R* is the universal gas constant; and *T* is temperature.

At the same time, the influence of the number of crystallization centers *I_c_* introduced during the application of nano-additives on the increase in the hydration rate ν results in an increase in the volume increment of the new phase (an increase in the degree of cement hydration) *dV* per unit time τ according to the expression [54,67].

(6) $$dV=\frac{4\pi I_{c}}{3}\left[V_{0}-V\left(\tau \right)\right]v^{3}{\left(\tau_{0}-\tau \right)}^{3}d\tau$$

In the general case, the structure-forming participation and modifying effect of nanosized modifiers can be the result of the following interrelated mechanisms [68,69]:

(1) A mechanism that provides an increase in the packing density of the system for the addition of dispersed particles, a decrease in its total porosity, and a change in the structure of the porosity of the material—the nanosized particles present in the system are able, by increasing the volume of adsorption and chemisorption-bound water, to reduce the volume of capillary-bound and free water—lead to change in the rheological properties of the cement paste and concrete mixture, to increase their viscosity and plastic strength;
(2) The mechanism associated with the catalytic role of nanosized particles as crystallization centers with the corresponding effect of lowering the energy threshold of this process and accelerating it;
(3) The mechanism of zoning the hardening structure by nanosized particles (microvolumes of the hardening structure are in the field of energy, thermodynamic influence of individual nanosized particles, which may be accompanied by the formation of an organized structure as a system of crystallites from hydrated phases);
(4) The mechanism associated with the possibility of the direct chemical participation of nanosized particles in the heterogeneous processes of the phase formation of hydrate compounds (this possibility is determined both by a substantial feature, the chemical and mineralogical compositions of particles, and by increased values of their specific surface area and specific surface energy) [54].

It is clear that the measure of implementation of these mechanisms of the nano-modification of the structure of the cement stone and their effectiveness was determined by the type, characteristics, dosage, and methods of introducing nanosized particles into the system. The implementation of the first mechanism was determined by the following interrelated factors related to the characteristics of additives: size, morphology, surface area, specific surface energy of nanosized particles, and their dosage. With a decrease in the size of nanosized particles, their surface area and specific surface energy related to the mass of particles increases, which not only fills the micropores, but also significantly reduces the amount of capillary-bound and free water, compacting the system. From this point of view, nano-modifiers of various substantive varieties, having a size of no more than 20 nm, of a spherical or tubular structure, capable of not only adsorption, but also chemisorption-binding water, are the most effective. The catalytic mechanism is realized at the stage of colloidation, nucleation, and phase formation, when nanosized particles act as crystalline seeds and crystallization centers. The most important factors in the implementation of this mechanism, depending on the properties of additives, are the following: the substance of nanosized particles and their size, which determine the duration of the mechanism, as well as the concentration of nanosized particles per unit volume of the hardening system. It should be pointed out that small nanosized particles (less than 10–20 nm) related to the minerals of the cement system in their crystallochemical structure can play the role of crystallization centers only for a very short time. Thus, the studies [68] found that the presence of nanosized silica particles with a diameter of 5–20 nm in a hardening system was observed only in the initial periods of hardening (8–24 h); then they were not fixed. This was due to their extremely high chemical activity and ability to participate in reactions, probably also by the topochemical mechanism. Nanosized particles that are chemically inactive with respect to cement systems, for example, carbon nanoparticles of a spherical and tubular structure, are observed in the material for a long time [54].

The material structure zoning mechanism is mainly determined by the specific surface energy of nanosized particles, which, in turn, is a function of particle size and their specific surface area. According to the calculations, the volume of space that is energetically zoned by one nanoparticle with a size of 5–20 nm is not only comparable to its own volume, but can also exceed it by a factor of 2–3. From this point of view, a decrease in the size of the nanoparticles makes it possible not only to significantly saturate the microvolumes of the material with energy, but also to reduce the dosage of nanosized particles, which favorably affects the economic side of the issue of their use in concrete technology. The chemical mechanism can be realized only under the condition that the composition of particles substantially corresponds to the products of hydration of cement minerals, since this is connected with their direct participation in the chemical reactions of the formation of a new phase. It is on this basis that it is preferable to modify the structure of cement stone with nanosized particles of calcium hydrosilicates, calcium hydrosulfoaluminates, chrysotile, and silica [54].

## 5. Conclusions

The existing ideas were developed from the point of view of theory and practice concerning the processes of cement gel formation when creating nano-modified high-strength concretes with nano-modifying additives.

The main scientific results of the study are as follows.

(1) The most rational method for the nano-modification of high-strength concretes was chosen through additional research in terms of optimization. The effective time of grinding microsilica to nanosilica was 12 h;
(2) A complex nano-modifier containing nanosilica, superplasticizer, hyperplasticizer and sodium sulfate was developed;
(3) The most significant influence on the strength characteristics of high-strength fine-grained concretes was exerted by the following factors: dosage of nanosilica; dosage of superplasticizer; and water–cement ratio;
(4) The most effective combination of the four considered factors was obtained: nanosilica content was 4% by weight of cement; the content of the superplasticizer additive was 1.4% by weight of cement; the content of the hyperplasticizer additive was 3% by weight of cement; and water–cement ratio was 0.33. The maximum difference in strength characteristics, in comparison to other combinations, ranged from 45% to 57%, depending on the type of strength;
(5) From the point of view of the structure formation of the cement composites, the developed nano-modifier allowed us to interfere and control the process of formation of new crystallization centers due to the active interaction between nanosilica particles, provided that these particles were rationally dosed, which formed new crystallization centers in the concrete body and contributed to the formation of a denser packing of particles that developed a more advanced structure. The effect was proven not only at the level of physically bound water, but also on the process of cement gel formation due to the appearance of new nano-modified crystallization centers.

Comprehensively assessing the achieved effect and taking into account the change in the cost of the material due to its nano-modification, reducing defects, and improving the quality and efficiency of construction as a whole, according to preliminary estimates, the cost of construction for improved concrete will decrease by about 10%.

The planned, promising directions for the development of research are the theoretical, experimental, and analytical tests of the effectiveness of the proposed ideas in relation to other nanomodifiers.

## Figures and Tables

**Table 1:** Characteristics of cement.

Property	Value	Standard
Specific surface, cm^2^/g	3253	GOST 30744-2001 “Methods of testing using polyfraction standard sand”
Normal density, %	26.5	
Density, kg/m^3^	3146	
Setting time, hour–min.		
- Start	2–25	
- End	3–45	
Compressive strength at 28 days, MPa	55.8	

**Table 2:** Chemical and mineralogical compositions of cement.

Element	Proportion, %
**Chemical composition**	
SiO_2_	21.03
Al_2_O_3_	5.03
Fe_2_O_3_	4.06
MgO	2.03
CaO	62.53
SO_3_	2.44
TiO_3_	0.064
LOI	2.2
Na_2_O	0.22
K_2_O	0.38
Chlorine ion CI¯	0.016
**Mineralogical composition**	
C_3_S	68
C_2_S	14
C_3_A	7
C_4_AF	11

**Table 3:** Characteristics of quartz sand.

Indicator Title		Value					
Grain Composition of Sand	Sieve Size, mm	2.5	1.25	0.63	0.315	0.16	<0.16
	Private Balances, %	18.6	12.5	24.6	31.6	9.3	3.1
	Total Balances, %	18.6	31.1	55.7	87.3	96.6	
Size modulus		2.9					
Content of dust and clay particles, %		0.85					
True grain density, kg/m^3^		2692					
Bulk density, kg/m^3^		1475					
Sand class by grain composition		1					
Voidness of sand, %		45					

**Table 4:** Technical description of MELFLUX 1641 F additive.

Indicator	Value
Delivery form	Powder
Color	Yellowish
Bulk density, g/L	480
pH	8.0
Storage temperature	from +5 °C to +35 °C

**Table 5:** Technical description of the MC-PowerFlow additive.

Indicator	Indicator Value
Supplement base	Polycarboxylate
Additive color	Brown liquid
Density, g/cm^3^	1.3
pH	6.5
Storage temperature	From +5 °C to +35 °C

**Table 6:** Technical description of sodium sulfate.

Indicator	Value
Appearance	Free-flowing white powder
Mass fraction of sodium sulfate, (Na_2_SO_4_), %	99.5
Mass fraction of water-insoluble residue, %	0.2
Mass fraction of chlorides in terms of sodium chloride (NaCl), %	0.2
Mass fraction of water, %	0.1
Indicator of activity of hydrogen ions of an aqueous 1% solution of sodium sulfate	8.2

**Table 7:** Parameters for optimizing the composition of the complex nano-modifier and their boundary values.

Optimization Parameter	Physical Meaning of Optimization Parameters
$R_{b,cub}$	Ultimate compressive strength of concrete samples at 28 days
$R_{b}$	Ultimate strength in axial compression of concrete samples at 28 days
$R_{btb}$	Ultimate tensile strength in the bending of concrete specimens aged 28 days
$R_{bt}$	Axial tensile strength of concrete samples at 28 days

**Table 8:** Values of variation factors.

Factor Code	The Physical Meaning of the Factor	Level of Variation					Interval of Variation δ
		−2	−1	0	1	+2	
X_1_	The content of nanosilica, % by weight of cement	2.5	3	4	5	5.5	1 and 0.5
X_2_	Additive content MELFLUX 1641 F, % by weight of cement	0.4	0.7	1.4	2.1	2.4	0.7 and 0.3
X_3_	The content of the additive “MC-PowerFlow”, % by weight of cement	1.5	2	3	4	4.5	1 and 0.5
X_4_	W/C ratio	0.30	0.31	0.33	0.35	0.36	0.02 and 0.01

**Table 9:** Results of determining the strength characteristics of high-strength concretes.

Num	Coded Factor Values				Natural Values Factor				$R_{b,cub}$	$R_{b}$	$R_{btb}$	$R_{bt}$
	X_1_	X_2_	X_3_	X_4_	X_1_	X_2_	X_3_	X_4_				
1	−1	−1	−1	−1	3	0.7	2	0.31	71.7	52.8	8.5	5.7
2	1	−1	−1	−1	5	0.7	2	0.31	77.3	57.1	9.7	5.9
3	−1	1	−1	−1	3	2.1	2	0.31	70.5	53.8	8.4	4.9
4	1	1	−1	−1	5	2.1	2	0.31	78.1	58.5	9.6	5.6
5	−1	−1	1	−1	3	0.7	4	0.31	72.8	55.6	8.3	5.0
6	1	−1	1	−1	5	0.7	4	0.31	79.6	52.1	8.8	5.6
7	−1	1	1	−1	3	2.1	4	0.31	70.8	54.1	8.5	5.3
8	1	1	1	−1	5	2.1	4	0.31	77.8	58.3	8.7	5.6
9	−1	−1	−1	1	3	0.7	2	0.35	72.4	55.3	8.5	5.3
10	1	−1	−1	1	5	0.7	2	0.35	69.8	52.4	8.9	4.9
11	−1	1	−1	1	3	2.1	2	0.35	68.7	51.5	8.8	4.8
12	1	1	−1	1	5	2.1	2	0.35	71.9	54.9	8.5	5.5
13	−1	−1	1	1	3	0.7	4	0.35	68.8	51.6	8.4	4.8
14	1	−1	1	1	5	0.7	4	0.35	71.3	53.5	9.2	5.0
15	−1	1	1	1	3	2.1	4	0.35	72.3	54.2	8.2	5.1
16	1	1	1	1	5	2.1	4	0.35	74.8	56.4	8.7	5.2
17	−2	0	0	0	2.5	1.4	3	0.33	67.0	52.3	9.9	4.7
18	2	0	0	0	5.5	1.4	3	0.33	76.9	57.7	9.1	5.4
19	0	−2	0	0	4	0.4	3	0.33	68.7	50.5	8.8	4.8
20	0	2	0	0	4	2.4	3	0.33	72.3	54.2	8.4	5.1
21	0	0	−2	0	4	1.4	1.5	0.33	82.1	61.6	11.8	5.7
22	0	0	2	0	4	1.4	4.5	0.33	75.8	58.9	11.6	5.3
23	0	0	0	−2	4	1.4	3	0.30	73.6	55.2	11.7	5.2
24	0	0	0	2	4	1.4	3	0.36	69.8	52.4	11.4	4.9
25	0	0	0	0	4	1.4	3	0.33	97.8	73.4	11.8	7.2
26	0	0	0	0	4	1.4	3	0.33	95.7	71.4	11.9	7.2
27	0	0	0	0	4	1.4	3	0.33	96.4	72.3	11.5	7.3
28	0	0	0	0	4	1.4	3	0.33	94.8	70.5	11.4	7.4
29	0	0	0	0	4	1.4	3	0.33	97.6	73.2	11.7	7.3
30	0	0	0	0	4	1.4	3	0.33	98.4	73.8	11.8	7.2
31	0	0	0	0	4	1.4	3	0.33	95.1	72.3	11.4	7.1

## Data Availability

The study did not report any data.
